# Effect of CPB glucose levels on inflammatory response after pediatric cardiac surgery

**DOI:** 10.1186/s12872-022-02667-w

**Published:** 2022-05-14

**Authors:** Zhi-Hua Zeng, Xin-Yi Yu, Xiao-Cheng Liu, Zhi-Gang Liu

**Affiliations:** Department of Cardiovascular Surgery, TEDA International Cardiovascular Hospital, Chinese Academy of Medical Sciences & Graduate School of Peking Union Medical College, No.61, the 3rd Ave, TEDA, Tianjin, 300457 China

**Keywords:** Cardiopulmonary bypass, Blood glucose, Severe SIRS, Association

## Abstract

**Background:**

Systemic inflammatory response syndrome (SIRS) is a common complication after cardiac surgery. There are no definite optimal glycemic threshold for pediatric patients receiving open-heart surgery with CPB. The study aimed to investigate the optimal cardiopulmonary bypass (CPB) glucose in patients undergoing cardiac surgery.

**Methods:**

We enrolled children with congenital heart disease who underwent surgical repair between June 2012 and December 2020. We included only patients who underwent cardiac surgery with CPB. The primary outcome was severe SIRS. A two-piece-wise regression model was applied to examine threshold effect of CPB glucose on severe SIRS.

**Results:**

A total of 7350 patients were enrolled in the present study, of whom 3895 (52.99%) are female. After potential confounders were adjusted, non-linear relationship was detected between CPB glucose and severe SIRS, whose turning point was 8.1. With CPB glucose < 8.1 mmol/L, the estimated dose–response curve was consistent with a horizontal line. However, the prevalence of severe SIRS increased with increasing glucose up to the turning point (Glucose > 8.1 mmol/L); the odds ratio (OR) of the Glucose was 1.35 (95% CI 1.21, 1.50).

**Conclusions:**

The present study indicates the association of CPB glucose with inflammatory response after pediatric cardiac surgery. The patients might have the best outcomes with the optimal CPB glucose no more than 8.1 mmol/L.

**Supplementary Information:**

The online version contains supplementary material available at 10.1186/s12872-022-02667-w.

## Background

Congenital heart disease (CHD) is the most common birth defect occurring in approximately 1% of all live births and affecting millions of individuals internationally [1]. Although surgical techniques had achieved massive breakthroughs, postoperative morbidity and mortality among infants and young children remain relatively high [2]. Systemic inflammatory response syndrome (SIRS) was a frequent complication after pediatric congenital heart surgery; it affects nearly one third of children and prolongs PICU stay significantly [3]. Therefore, identifying modifiable risk factors that could lower the incidence of perioperative SIRS is important for sustained improvement in clinical outcomes of these patients.

Perioperative hyperglycemia is related to cardiopulmonary bypass (CPB) [4]. Several clinical studies suggest that hyperglycemia is associated with postoperative morbidity in patients who undergo cardiac surgery [5–7]. The perioperative period for congenital heart surgery can be challenging because of the systemic inflammatory response and endocrine metabolic stress associated with these procedures [8].

Improved glycemic control at initiation of CPB in adult patients undergoing cardiac surgery was associated with reduced 30-day mortality [9]. Nevertheless, there are no definite optimal glycemic threshold or reference interval for pediatric patients receiving open-heart surgery with CPB. In this study, we aimed to investigate the association of CPB glucose with severe SIRS in pediatric patients receiving open-heart surgery with CPB. And according to this glycemic threshold, we can reduce the incidence of SIRS and related complications by optimizing glycemic management during CPB.

## Methods

### Research population

This respective cohort study was conducted in pediatric patients who underwent cardiac surgery at TEDA International Cardiovascular Hospital. We included all patients who underwent cardiac surgery with arrested-heart CPB between June 2012 and December 2020. We excluded patients undergoing a cardiac procedure on a beating heart or required preoperative renal-replacement therapy, mechanical ventilatory support, or mechanical circulatory support. We also excluded those who had missing CPB glucose data or outcome. The present study was approved by the Ethics Committee (Internal Review Board) of TEDA International Cardiovascular Hospital. All the procedures performed in this study involving human participants were conducted in accordance with the Declaration of Helsinki (as revised in 2013). All data collection was done anonymously. The requirement of personal consent for this retrospective analysis was waived by the Ethics Committee (Internal Review Board) of TEDA International Cardiovascular Hospital, so there is no confusion regarding prospective consent.

### Research exposure

All blood glucose measurements during cardiopulmonary bypass were collected.

In this study, maximum CPB arterial glucose values were retrieved from a local online hospital information system for analysis and further confirmed by independent manual examination of extracorporeal circulation records.

### Research covariates

For each patient, those baseline and clinical characteristics, including Gender, Age of surgery (month), Age category of surgery, Body surface area (m^2^), BMI (kg/m^2^), Surgery year, Preoperative hemoglobin, Residence altitude, Hemodynamic pathology, Extracardiac malformations, Genetic anomalies, Clinical pathway implementation, Pulmonary arterial hypertension (PAH), Aorta crossclamp time (min), Red Cell need (U) during CPB, Steroids need, Glucose infusion, Insulin need, Aristotle complexity score and level were collected.

### Research outcome

The primary outcome variable was severe systemic inflammatory response syndrome (SIRS), which we defined as the time of onset of SIRS from admission to the intensive care unit (ICU), postoperative day 5, or discharge [10]. According to the definition of pediatric SIRS or sepsis, as well as the diagnostic criteria used clinically in our center, severe SIRS is defined by the satisfaction of four criteria below: (1) Body temperature over 38 or under 36 degrees Celsius; (2) Mean heart rate > 2 standard deviations (SD) beyond normal for age; (2) Mean respiratory rate > 2 SD above normal for age; (3) Elevated or reduced age-specific leukocyte count or > 10% immature neutrophils [3, 11, 12]. Secondary outcomes included SIRS length of mechanical ventilation, ICU stay, and all-cause mortality at 30 days postoperatively.

### Statistical analysis

All analyses were performed using EmpowerStats (http://www.empowerstats.com). Baseline and clinical materials were grouped by glucose (8.1 mmol/L). Categorical variables are presented as percentages. Continuous variables are reported as medians with interquartile range (IQR). Comparisons between groups were performed using χ^2^ testing for categorical variables and Kruskal–Wallis testing for continuous variables. Univariate linear regression model was used to evaluate the associations between CPB glucose and severe SIRS. We used used generalized additive model (GAM) to identify the non-linear relationship between glucose and severe SIRS. And we applied a two-piece-wise regression model to examine threshold effect of CPB glucose on severe SIRS. To examine the cumulative incidence of severe SIRS by age at surgery, we used Kaplan–Meier estimates, using age as the time scale [13]. *P*-value < 0.05 was defined as statistically significant.

## Results

### Patient characteristics and primary outcome

**Fig. 1:**
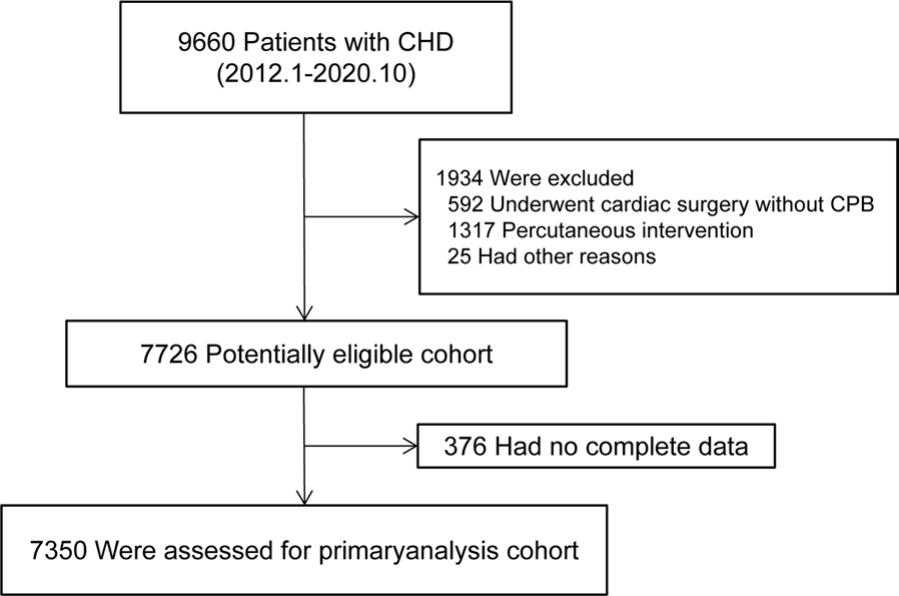
The selected study patients. CHD, Congenital heart diseases; CPB, cardiopulmonary bypass

A total of 7350 patients were enrolled in the present study (Fig. 1), of whom 5821 (78.20%) patients had CPB glucose less than 8.1 mmol/L. 3895 (52.99%) are female, 37.5 months (IQR 18.91–69.83) was the median age at the time of surgery, and the median glucose during CPB was 6.40 (IQR 5.3–7.8) mmol/L. Baseline characteristics of patients with low (< 8.1 mmol/L) and high (> 8.1 mmol/L) glucose are shown in Table 1. Low glucose patients were older and congenital heart disease clinical pathways were more frequently implemented (Table 1).

**Table 1:** Characteristics and results between low and high glucose patients

	All patients (N = 7350)	Low glucose patients (N = 5821)	High glucose patients (N = 1529)	*P*-value
*Sociodemography*				
Age of surgery, month	37.53 (18.91–69.83)	40.23 (20.70–72.00)	28.73 (12.77–57.37)	< 0.001
*Gender*				
Female	3895 (52.99%)	3096 (53.19%)	799 (52.26%)	0.517
Male	3455 (47.01%)	2725 (46.81%)	730 (47.74%)	
*Surgery year*				< 0.001
2012–2014	2713 (36.91%)	1976 (33.95%)	737 (48.20%)	
2015–2017	2527 (34.38%)	1999 (34.34%)	528 (34.53%)	
2018–2020	2110 (28.71%)	1846 (31.71%)	264 (17.27%)	
*Age category of surgery*				0.018
Infants	2127 (28.94%)	1491 (25.61%)	636 (41.60%)	
Toddlers and preschoolers	3526 (47.97%)	2902 (49.85%)	624 (40.81%)	
School age	1415 (19.25%)	1187 (20.39%)	228 (14.91%)	
Teenagers	282 (3.84%)	241 (4.14%)	41 (2.68%)	
Body surface area, m^2^	0.59 (0.47–0.75)	0.60 (0.49–0.77)	0.52 (0.42–0.68)	< 0.001
BMI, kg/m^2^	15.19 (14.07–16.53)	15.18 (14.07–16.49)	15.27 (14.05–16.68)	0.370
Preoperative hemoglobin	128.00 (119.00–136.00)	128.00 (119.00–136.00)	128.00 (117.00–139.00)	0.456
*Residence altitude*				0.022
Low altitude	5038 (68.54%)	3953 (67.91%)	1085 (70.96%)	
High altitude	2312 (31.46%)	1868 (32.09%)	444 (29.04%)	
*Inpatient Information*				
*Hemodynamic pathology*				< 0.001
Non cyanotic	6262 (85.20%)	5025 (86.33%)	1237 (80.90%)	
Cyanotic	1088 (14.80%)	796 (13.67%)	292 (19.10%)	
*Extracardiac malformations*				0.746
Absence	7053 (95.96%)	5588 (96.00%)	1465 (95.81%)	
Presence	297 (4.04%)	233 (4.00%)	64 (4.19%)	
*Genetic anomalies*				0.009
Non	6964 (94.74%)	5495 (94.40%)	1469 (96.08%)	
Presence	386 (5.13%)	326 (5.60%)	60 (3.92%)	
*Clinical pathway implementation*				< 0.001
Non	1299 (17.67%)	877 (15.07%)	422 (27.60%)	
Presence	6051 (82.33%)	4944 (84.93%)	1107 (72.40%)	
*PAH*				< 0.001
Non	4585 (62.38%)	3558 (61.12%)	1027 (67.17%)	
Presence	2765 (37.62%)	2263 (38.88%)	502 (32.83%)	
*Surgical factors*				
Aorta crossclamp time, min	34.00 (23.00–52.00)	32.00 (22.00–45.00)	51.00 (32.00–89.00)	< 0.001
Red cell need, U	1.00 (0.00–2.00)	1.00 (0.00–1.00)	1.00 (1.00–2.00)	< 0.001
*Steroids need*				< 0.001
No	7052 (95.95%)	5674 (97.47%)	1378 (90.12%)	
Yes	298 (4.05%)	147 (2.53%)	151 (9.88%)	
*Glucose infusion*				0.072
No	7179 (97.67%)	5695 (97.84%)	1484 (97.06%)	
Yes	171 (2.33%)	126 (2.16%)	45 (2.94%)	
*Insulin need*				< 0.001
No	6712 (91.32%)	5821 (100.00%)	891 (58.27%)	
Yes	638 (8.68%)	0 (0.00%)	638 (41.73%)	
Aristotle complexity score	6.00 (6.00–7.00)	6.00 (6.00–7.00)	6.00 (6.00–7.00)	0.127
*Aristotle complexity level*				0.003
Level 1	1197 (16.29%	943 (16.20%)	254 (16.61%)	
Level 2	5020 (68.30%)	4024 (69.13%)	996 (65.14%)	
Level 3	1005 (13.67%)	762 (13.09%)	243 (15.89%)	
Level 4	128 (1.74%)	92 (1.58%)	36 (2.35%)	
*Exposure*				
Glucose, mmol/L	6.40 (5.30–7.80)	5.90 (5.10–6.80)	9.60 (8.70–11.10)	< 0.001
*Outcomes*				
*Primary outcome*				
Severe SIRS	1600 (21.77%)	1003 (17.23%)	597 (39.05%)	< 0.001
*Secondary outcome*				
SIRS	5637 (76.69%)	2937 (75.40%)	2700 (78.15%)	0.005
Length of mechanical ventilation, hours	3.15 (2.10–5.20)	3.00 (2.00–4.44)	4.40 (2.55–20.27)	< 0.001
ICU stay, hours	22.08 (19.00–39.58)	21.92 (18.83–23.83)	22.75 (20.00–46.75)	< 0.001

BMI, Body mass index; PAH, Pulmonary arterial hypertension; SIRS, systemic inflammatory response syndrome; ICU, intentive care unit

*Data are n (%) or median (IQR) other indicated mean (SD)

Low glucose patients had a higher frequency of, genetic anomalies and pulmonary hypertension, but had a lower frequency of hemodynamic pathology in comparison to high glucose patients (Table 1). There were no significant differences between the two groups in terms of extracardiac malformations (Table 1). The overall incidence of severe SIRS was 21.77%, and the incidence of severe SIRS in low glucose patients and high glucose patients was 17.23% and 39.05% (Table 1).

### Secondary outcome

High glucose patients had a significantly longer postoperative length of mechanical ventilation and ICU stay in comparison to low glucose patients (*P* < 0.01) and a higher incidence of SIRS (*P* = 0.005) and inpatient mortality (*P* < 0.001).

### Univariate analysis

The results of univariate analysis were shown in Table 2. The results of univariate analysis showed that age of surgery, body surface area, preoperative hemoglobin, cyanotic, clinical pathway implementation, pulmonary arterial hypertension, aorta crossclamp time, red cell need, steroids need, insulin need, aristotle complexity score, aristotle complexity level were correlated with higher severe SIRS. We also found that gender, age category of surgery, BMI, residence altitude, extracardiac malformations, genetic anomalies, were not associated with severe SIRS.

**Table 2:** The results of univariate analysis

	Statistics	Effect size (β)	*P*-value
Age of surgery, month	37.53 (18.91–69.83)	1.01 (1.01, 1.01)	< 0.001
*Age category of surgery*			0.018
Infants	2127 (28.94%)	1.0	
Toddlers and preschoolers	3526 (47.97%)	1.37 (1.17, 1.61)	< 0.0001
School age	1415 (19.25%)	8.63 (7.30, 10.20)	< 0.0001
Teenagers	282 (3.84%)	0.85 (0.56, 1.27)	0.4169
*Gender*			
Female	3895 (52.99%)	1.0	
Male	3455 (47.01%)	1.01 (0.91, 1.13)	0.8256
*Surgery year*			
2012–2014	2713 (36.91%)	1.0	
2015–2017	2527 (34.38%)	0.98 (0.86, 1.11)	0.7312
2018–2020	2110 (28.71%)	0.63 (0.54, 0.72)	< 0.0001
Body surface area, m^2^	0.59 (0.47–0.75)	4.26 (3.51, 5.17)	< 0.001
BMI, kg/m^2^	15.19 (14.07–16.53)	1.01 (0.99, 1.04)	0.3206
Preoperative hemoglobin	128.00 (119.00–136.00)	1.02 (1.02, 1.02)	< 0.0001
*Residence altitude*			
Low altitude	5038 (68.54%)	1.0	
High altitude	2312 (31.46%)	1.07 (0.95, 1.20)	0.2811
*Hemodynamic pathology*			
Non cyanotic	6262 (85.20%)	1.0	
Cyanotic	1088 (14.80%)	1.63 (1.41, 1.88)	< 0.0001
*Extracardiac malformations*			
Absence	7053 (95.96%)	1.0	
Presence	297 (4.04%)	1.09 (0.83, 1.44)	0.5328
*Genetic anomalies*			
Non	6964 (94.74%)	1.0	
Presence	386 (5.13%)	0.92 (0.71, 1.19)	0.5242
*Clinical pathway implementation*			
Non	1299 (17.67%)	1.0	
Presence	6051 (82.33%)	0.66 (0.57, 0.75)	< 0.0001
*PAH*			
Non	4585 (62.38%)	1.0	
Presence	2765 (37.62%)	0.70 (0.62, 0.78)	< 0.0001
Aorta crossclamp time, min	34.00 (23.00–52.00)	1.01 (1.01, 1.01)	< 0.001
Red cell need, U	1.00 (0.00–2.00)	0.93 (0.88, 0.99)	0.0157
*Steroids need*			< 0.001
No	7052 (95.95%)	1.0	
Yes	298 (4.05%)	1.81 (1.41, 2.32)	< 0.0001
*Glucose infusion*			
No	7179 (97.67%)	1.0	
Yes	171 (2.33%)	0.96 (0.66, 1.39)	0.8184
*Insulin need*			
No	6712 (91.32%)	1.0	
Yes	638 (8.68%)	9.33 (7.82, 11.12)	< 0.0001
Aristotle complexity score	6.00 (6.00–7.00)	1.13 (1.09, 1.18)	< 0.0001
*Aristotle complexity level*			
Level 1	1197 (16.29%	1.0	
Level 2	5020 (68.30%)	1.24 (1.05, 1.46)	0.0096
Level 3	1005 (13.67%)	1.78 (1.45, 2.18)	< 0.0001
Level 4	128 (1.74%)	3.18 (2.17, 4.66)	< 0.0001
Glucose, mmol/L	6.40 (5.30–7.80)	1.32 (1.29, 1.35)	< 0.001

### Association of continuous glucose with outcome

**Fig. 2:**
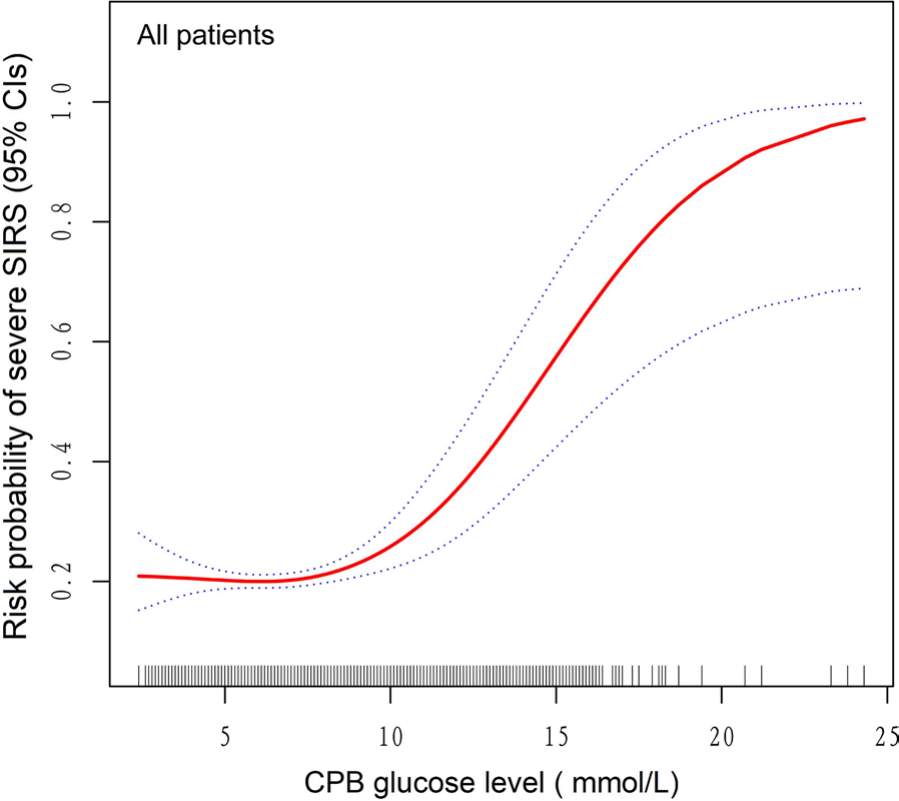
The relationship between CPB glucose and risk probability of severe SIRS. A gradual J-shaped risk curve among total patients. Red dotted lines represent the spline plots of CPB glucose and blue dotted lines represent the 95% CIs of the spline plots. Adjusted for Gender, Age of surgery (month), Age category of surgery, Body surface area (m^2^), BMI (kg/m^2^), Surgery year, Preoperative hemoglobin, Residence altitude, Hemodynamic pathology, Extracardiac malformations, Genetic anomalies, Clinical pathway implementation, pulmonary arterial hypertension (PAH), Aorta crossclamp time (min), Red Cell need (U), Steroids need, Glucose infusion, Insulin need, Aristotle complexity score, Aristotle complexity level

Because glucose was continuous variable, the analyses of non-linear relationship are necessary. In the present study (Fig. 2), we found that the relationship between glucose and severe SIRS was non-linear (after adjusting gender, age of surgery, age category of surgery, body surface area, BMI, surgery year, preoperative hemoglobin, residence altitude, hemodynamic pathology, extracardiac malformations, genetic anomalies, clinical pathway implementation, pulmonary arterial hypertension, aorta crossclamp time, red cell need, steroids need glucose infusion, insulin need, aristotle complexity score, aristotle complexity level).

By two-piece-wise linear regression model, we calculated the turning point was 8.1. With a Glucose < 8.1 mmol/L, the estimated dose–response curve was consistent with a horizontal line. However, the prevalence of severe SIRS increased with increasing glucose up to the turning point (Glucose > 8.1 mmol/L); the *odds ratio* (*OR*) of the Glucose was 1.35 (1.21, 1.50) < 0.0001 (Table 3). The threshold of glucose would result in a risk probability of roughly0.37 or greater for severe SIRS (Table 3).

**Table 3:** Threshold effect analysis of CPB glucose on severe SIRS using two-piece-wise regression model

	Severe SIRS
	Adjusted β/OR (95% CI) *p*-value
*Model I*	
One line slop	1.08 (1.03, 1.13) 0.0008
*Model II*	
Turning point (K)	8.1
Glucose < 8.1 mmol/L	0.98 (0.92, 1.04) 0.4151
Glucose > 8.1 mmol/L	1.35 (1.21, 1.50) < 0.0001
LRT test	< 0.001^#^

LRT test, Logarithmic likelihood ratio test

^#^Model II is significant different from Model I

Adjusted: Gender, Age of surgery (month), Age category of surgery, Body surface area (m^2^), BMI (kg/m^2^), Surgery year, Preoperative hemoglobin, Residence altitude, Hemodynamic pathology, Extracardiac malformations, Genetic anomalies, Clinical pathway implementation, pulmonary arterial hypertension (PAH), Aorta crossclamp time (min), Red cell need (U), Steroids need, Glucose infusion, Insulin need, Aristotle complexity score, Aristotle complexity level

### Severe SIRS-free probability by age

**Fig. 3:**
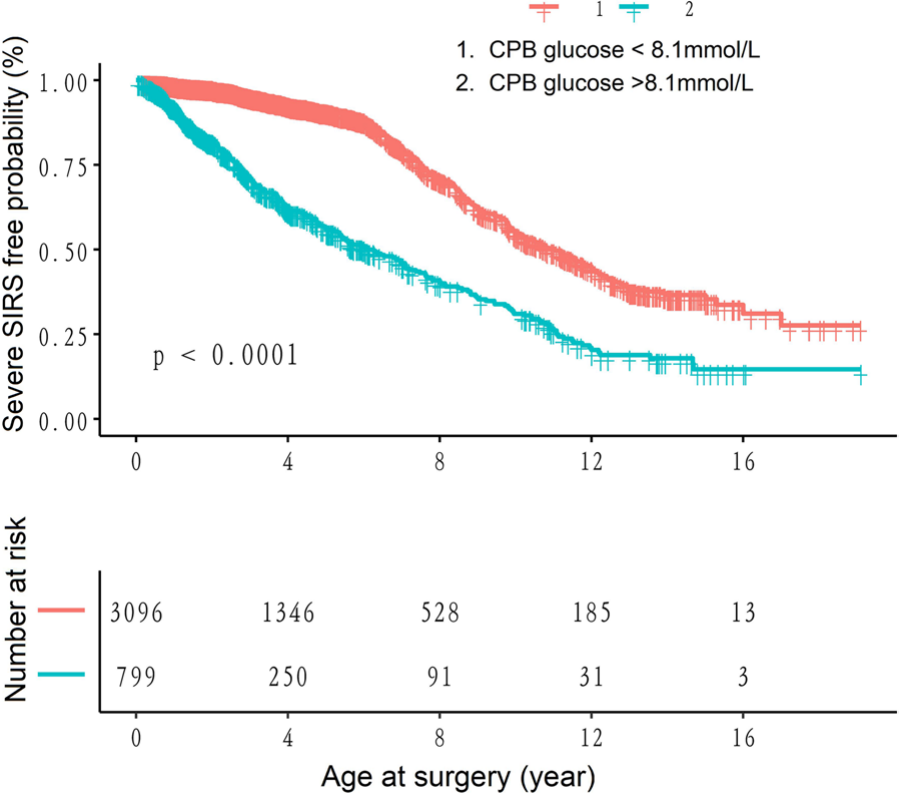
Severe SIRS-free probability with increasing age by glucose category for overall patients. The figure represents the severe SIRS-free probability by age separately for all patients depending on glucose category

Figure 3 shows the severe SIRS-free probability by age separately for overall patients. For all patients, this age gradient was steeper for those with glucose > 8.1 mmol/L, and the patients with glucose < 8.1 mmol/L have the lower risk probability (*P* < 0.001) in overall patients (Fig. 3).

## Discussion

This study examines the association between CPB glucose and postoperative severe SIRS in the pediatric cardiac surgical population. These data show that there is an association between an CPB glucose level of greater than 8.1 mmol/L and the development of a postoperative severe SIRS. In our study, Non-linear relationship was detected between CPB glucose and severe SIRS, whose turning point was 8.1 mmol/L. The probability of postoperative severe SIRS increased with elevated CPB glucose up to the turning point (Glucose = 8.1 mmol/L). Using the overall rate of severe SIRS as reference, CPB glucose threshold of SIRS might lower 8.1 mmol/L, which conduce to management of extracorporeal circulation.

Previous studies have suggested that the optimal postoperative glycemic range in children undergoing complex congenital heart surgery was likely to be 6.1–7 mmol/L [9]. Perioperative mean glucose ≤ 8.3 mmol/L may decreased adverse events in infants receiving open cardiac surgery [14]. The recommended threshold is remarkably near to the level at which our data showed significance for the development of postoperative severe SIRS. In the present study (Fig. 2), we found that the relationship between CPB glucose and severe SIRS was non-linear (after adjusting gender, age of surgery, age category of surgery, body surface area, BMI, surgery year, preoperative hemoglobin, residence altitude, hemodynamic pathology, extracardiac malformations, genetic anomalies, clinical pathway implementation, pulmonary arterial hypertension, aorta crossclamp time, red cell need, steroids need, glucose infusion, insulin need, aristotle complexity score, aristotle complexity level). This indicated that CPB glucose was related to severe SIRS after pediatric cardiac surgery. Overall, there was a gradual J-shaped risk curve among total patients (Fig. 2). Previous studies have examined the effect of hyperglycemia on morbidity and mortality in the pediatric population [15]. Intraoperative hyperglycemia was also related to worse hospital outcomes after cardiac surgery, including death [16]. A study showed that strict glycemic control significantly decreased morbidity and mortality in critically ill children [17]. Strict intraoperative and postoperative glycemic control protects the myocardium and reduces the inflammatory response in neonatal cardiac surgery [8].

In the current study, The probability of severe SIRS significantly increased with elevated CPB glucose up to the turning point (Glucose = 8.1 mmol/L). Furthermore, We examined the cumulative incidence of severe SIRS by age at surgery and found that the patients with glucose < 8.1 mmol/L had the lower risk probability (*P* < 0.001) in overall patients (Fig. 3). These results highlighted that a significant take away maybe to maintain on CPB glucose < 8.1 mmol/L for all patients. Our study indicates that high glucose patients were associated with significantly greater longer postoperative ventilation and ICU stay, and a higher incidence of inpatient mortality in comparison with those low glucose patients, highlighting reasonable CPB glucose control for CHD children in the management of extracorporeal circulation.

### Limitations of the study

Our study has several potential limitations. The small set of variables available for covariate-adjusted analyses leaves the possibility of residual confounding. It is the highest arterial glucose that we selected for association analysis in our study that may not be comprehensive enough to reveal the clinical significance of glucose in postoperative inflammatory responses, so further study using the average arterial glucose may more reasonable. This study only studied blood glucose during extracorporeal circulation, and further studies should be conducted in conjunction with postoperative blood glucose.

## Conclusion

To the best of our knowledge, this is the first study to investigate the optimal CPB glucose in pediatric cardiac surgery. Our study provides evidence supporting that patients might have the best outcomes with the optimal CPB glucose no more than 8.1 mmol/L. These findings can promote to management of CPB glucose.

## Supplementary Information

**Additional file 1**. Data source.

## Data Availability

All data generated or analysed during this study are included in this published article and its Additional file 1.

## Abbreviations

CHD: Congenital heart disease
CPB: Cardiopulmonary bypass
SIRS: Systemic inflammatory response syndrome
BMI: Body mass index
PAH: Pulmonary arterial hypertension
IQR: Interquartile range
ICU: Intensive care unit
OR: Odds ratio
SD: Indicated mean
CI: Confidence Interval
